# Unveilling genetic profiles and correlations of biofilm-associated genes, quorum sensing, and antibiotic resistance in *Staphylococcus aureus* isolated from a Malaysian Teaching Hospital

**DOI:** 10.1186/s40001-024-01831-6

**Published:** 2024-04-22

**Authors:** Yun Li Chan, Chin Fei Chee, Soo Nee Tang, Sun Tee Tay

**Affiliations:** 1 Department of Medical Microbiology, Faculty of Medicine, Universiti Malaya, 50603 Kuala Lumpur, Malaysia; 2 Nanotechnology and Catalysis Research Centre, Universiti Malaya, 50603 Kuala Lumpur, Malaysia

**Keywords:** Biofilm production, MSSA, MRSA, Biofilm-associated gene, Antibiotic resistance

## Abstract

**Background:**

*Staphylococcus aureus* is a notorious multidrug resistant pathogen prevalent in healthcare facilities worldwide. Unveiling the mechanisms underlying biofilm formation, quorum sensing and antibiotic resistance can help in developing more effective therapy for *S. aureus* infection. There is a scarcity of literature addressing the genetic profiles and correlations of biofilm-associated genes, quorum sensing, and antibiotic resistance among *S. aureus* isolates from Malaysia.

**Methods:**

Biofilm and slime production of 68 methicillin-susceptible *S. aureus* (MSSA) and 54 methicillin-resistant (MRSA) isolates were determined using a a plate-based crystal violet assay and Congo Red agar method, respectively. The minimum inhibitory concentration values against 14 antibiotics were determined using VITEK® AST-GP67 cards and interpreted according to CLSI-M100 guidelines. Genetic profiling of 11 *S. aureus* biofilm-associated genes and *agr*/*sar* quorum sensing genes was performed using single or multiplex polymerase chain reaction (PCR) assays.

**Results:**

In this study, 75.9% (*n* = 41) of MRSA and 83.8% (*n* = 57) of MSSA isolates showed strong biofilm-forming capabilities. Intermediate slime production was detected in approximately 70% of the isolates. Compared to MSSA, significantly higher resistance of clindamycin, erythromycin, and fluoroquinolones was noted among the MRSA isolates. The presence of intracellular adhesion A (*icaA*) gene was detected in all *S. aureus* isolates. All MSSA isolates harbored the laminin-binding protein (*eno*) gene, while all MRSA isolates harbored intracellular adhesion D (*icaD)*, clumping factors A and B (*clfA* and *clfB*) genes. The presence of *agrI* and elastin-binding protein (*ebpS*) genes was significantly associated with biofilm production in MSSA and MRSA isolates, respectively. In addition, *agrI* gene was also significantly correlated with oxacillin, cefoxitin, and fluoroquinolone resistance*.*

**Conclusions:**

The high prevalence of biofilm and slime production among MSSA and MRSA isolates correlates well with the detection of a high prevalence of biofilm-associated genes and *agr* quorum sensing system. A significant association of *agrI* gene was found with cefoxitin, oxacillin, and fluoroquinolone resistance. A more focused approach targeting biofilm-associated and quorum sensing genes is important in developing new surveillance and treatment strategies against *S. aureus* biofilm infection.

**Supplementary Information:**

The online version contains supplementary material available at 10.1186/s40001-024-01831-6.

## Introduction

*Staphylococcus aureus* is one of the leading causes of severe bacterial infections which may lead to life-threatening conditions, including sepsis, pneumonia, endocarditis, osteomyelitis, and implant-associated diseases [1–3]. The emergence of antibiotic resistance in *S. aureus* has posed a significant impact on the treatment and infection control practices in hospitals worldwide [4, 5]. Methicillin-resistant *S. aureus* (MRSA), vancomycin-resistant *S. aureus* (VRSA) and vancomycin-intermediate *S. aureus* (VISA) are among the pathogens listed as “High” priority in the World Health Organization (WHO)’s priority pathogens list for research and development of new antibiotics [6].

The ability of *S. aureus* to resist antimicrobials is further enhanced by the strategy of biofilm formation. Currently available antibiotics cannot eradicate biofilms, especially of ESKAPE pathogens, which includes MRSA [7]. Several studies found no statistically significant difference in the biofilm formation among MRSA and MSSA strains [8, 9]. In contrary, a study reported that MRSA strains showed enhanced biofilm formation as compared to MSSA strains [10]. Staphylococcal polysaccharide intercellular adhesin (encoded by *icaABCD*) [11], collagen-binding protein (*cna*), fibrinogen binding protein (*fib*), elastin binding protein (*ebpS*), laminin binding protein (*eno*), fibronectin binding proteins A and B (*fnbA* and *fnbB*), and clumping factors A and B (*clfA* and *clfB*) [12] have been reported to play important roles in *S. aureus* adherence, which is the first step in biofilm production. The *icaABCD* operon is also known for its function in slime production [13].

Quorum sensing is a mechanism, whereby bacterial cells communicate and coordinate their behaviours based on population density [14]. The accessory gene regulator (*agr*) quorum-sensing system plays a key role in *S. aureus* pathogenesis, while the staphylococcal accessory regulator (*sarA*) gene is essential in controlling staphylococcal virulence factors [15]. Both *agr* and *sarA* quorum sensing genes have been reported to regulate *S. aureus* biofilm formation [15–18]. To date, four polymorphic *agr* types (*agrI*, *agrII*, *agrIII*, and *agrIV*) have been reported [19].

Previously, a high prevalence of *icaADBC* genes and varied occurrence of biofilm associated genes, i.e., *cna* (42.7–93%), *fib* (24.7–90%), *ebps* (11.1–100%), *fnbA* (0–100%) and *fnbB* (1.1–53.33%) have been reported in Malaysian *S. aureus* clinical isolates [20–22]. The *agr1* was the most prevalent type reported in Malaysian isolates of *S.aureus,* followed by *agrII* and *agrIII*; however, no *agrIV* was detected [21, 23]. Understanding differences in biofilm and slime production between MRSA and MSSA and the associated genetic elements contributes to a better understanding of the epidemiology and spread of *S. aureus* infections, further aiding in developing more targeted surveillance and treatment strategies. Hence, this study was performed to analyze biofilm and slime production of a collection of MSSA and MRSA clinical isolates and to investigate possible correlations between biofilm-associated genes and the *agr*/*sar* quorum sensing systems in relation to antibiotic resistance.

## Methods

### Collection of clinical isolates

A total of 68 MSSA and 54 MRSA isolates collected from patients attending Universiti Malaya Medical Centre (UMMC) from August 2020 to June 2022 were investigated in this study. The isolates were primarily collected from the blood (*n* = 38, 31.1%), and tissue (*n* = 36, 29.5%), followed by pus (*n* = 15, 12.3%), wound swab (*n* = 11, 9%), and lower respiratory tract (*n* = 18, 14.8%) (Additional file 1: Table S1). The identity of the isolates was confirmed using matrix*-*assisted laser desorption/ionization time-of-flight mass spectrometry (VITEK MS system, bioMérieux Clinical Diagnostics, France).

### Antibiotic susceptibility testing

The minimum inhibitory concentration values (MIC) of *S. aureus* against 14 antibiotics, i.e., clindamycin, penicillin, erythromycin, gentamicin, linezolid, oxacillin, rifampicin, cotrimoxazole, tetracycline, vancomycin, ciprofloxacin, levofloxacin, moxifloxacin, and cefoxitin were determined using VITEK® AST-GP67 card (bioMérieux Clinical Diagnostics, France), and interpreted according to the Clinical Laboratory Standards Institute (CLSI-M100) guidelines [24]. Methicillin susceptibility of the clinical isolates was determined using the CLSI disk–diffusion method with cefoxitin 30-µg disk and VITEK® AST-GP67 card. For 10 isolates with missing MIC data, vancomycin susceptibility testing was carried out using microbroth dilution method, as recommended by CLSI-M100 guidelines.

### Biofilm quantitation assay

Quantitation of *S. aureus* biofilm production was performed as described by Atshan et al. [22] and Stepanović et al. [25], with slight modifications. Briefly, 100 µl of bacterial suspension (adjusted to 1 × 10^6^ CFU/ml in Mueller Hinton broth containing 1% glucose) were seeded into each well of a sterile 96*-*well flat bottom microtitre plate (BIOFIL®, Guangzhou, China) and incubated at 37ºC for 24 h. After incubation, the wells were washed thrice, fixed with methanol, and stained using 0.1% (v/v) crystal violet (Cat. No: C6158, Sigma, USA). *S. aureus* ATCC® 29213™ (MSSA) and ATCC® 33591™ (MRSA) were used as biofilm-producing controls, while microtiter wells with no inoculum served as negative controls. The amount of biofilm was quantitated by measuring the absorbance of each well at 570 nm using a microplate reader (Tecan, Sunrise™, Swiss). Biofilm was graded into four categories as described by Moghadam et al. [26]: no biofilm (ODs ≤ ODc), weak (ODc ≤ ODs ≤ 2 × ODc), moderate (2 × ODc ≤ ODs ≤ 4 × ODc), and strong (4 × ODc < ODs). ODc and ODs represent the OD of the negative and the test isolates, respectively.

### Congo red agar assay for determination of slime production

Bacterial slime production was determined qualitatively as described by Freeman et al. [27] and Thilakavathy et al. [28]. Congo red agar was prepared using brain heart infusion (BHI) broth (37 g/L), sucrose (50 g/L), agar no.1 (10 g/L), and Congo red stain (0.8 g/L). Slime producers are expected to form black colonies with a dry, crystalline consistency, while non-slime producers form pink coloured colonies. Intermediate slime production is indicated by the growth of smooth blackish-red colonies. The positive and negative control strains included in the test were *Staphylococcus epidermidis* ATCC® 35984™ and *Staphylococcus hominis* ATCC® 35982™, respectively.

### Bacterial genomic DNA extraction

Genomic DNA was extracted from overnight cultures of *S. aureus* in Luria–Bertani broth, using either MasterPure™ Complete DNA and RNA Purification Kit (Lucigen, Middleton, WI, USA) or QIAamp DNA Mini Kit (Qiagen, Germany) following manufacturers’ instructions. Amplification of the 16S rRNA gene from the bacterial DNA extract was performed to rule out the possibility of having PCR inhibitors, using universal oligonucleotide primers (27F and 1492R) as described by Gumaa et al. [29].

### PCR detection of biofilm-associated genes

PCR profiling of *bap, cna, icaA,* and *icaD* genes was performed using singleplex PCR assays, while *ebpS*, *eno*, *fnbA*, *clfA*, *clfB*, *fib*, and *fnbB* genes were amplified using multiplex PCR assays as described by Tristan et al. and Vancraeynest et al. [30, 31]. The primers and PCR thermal cycling conditions are shown in Additional file 1: Table S2. s*arA* gene was amplified using *sar*AF and *sar*AR primers as described by Gowrishankar et al. [32]. Meanwhile, *agr* typing (types I–IV) was performed using primers and amplification conditions as described by Shopsin et al. [19]. The amplified products were then subjected to electrophoresis using 1% (w/v) agarose gel, pre-stained with nucleic acid staining dye (Bioteke Corporation, China). Sequence analyses were performed to confirm that correct genes were amplified.

### Statistical analysis

Paired sample *t* tests were used to compare biofilm and slime production between MSSA and MRSA isolates. Pearson’s Chi-square test was used to determine the correlation of antibiotic resistance with other parameters. Statistical analysis was performed using SPSS software version 20.0 (IBM, Armonk, USA). A *p* value of less than 0.05 was considered statistically significant.

## Results

### Antibiotic susceptibility profiling of *S. aureus* clinical isolates

MRSA isolates exhibited higher rates of resistance to erythromycin (53.7% vs 17.6%), ciprofloxacin (83.3% vs 2.9%), levofloxacin (83.3% vs 1.5%) and moxifloxacin (75.9% vs 0%), compared to MSSA isolates (Table 1). Clindamycin resistance was observed in 16.2% and 7.6% of MSSA and MRSA isolates, respectively, while inducible clindamycin resistance was detected in 23 (42.6%) MRSA isolates and 1 (1.5%) MSSA isolate. The MRSA MIC_90_s against clindamycin (0.5 vs 8 µg/ml), erythromycin (0.5 vs 8 µg/ml), gentamicin (0.5 vs 8 µg/ml), ciprofloxacin (0.5 vs 8 µg/ml), and levofloxacin (0.25 vs 8 µg/ml) were 16–32 folds higher than those of MSSA isolates (Additional file 1: Table S3). Meanwhile, all isolates exhibited high susceptibility towards linezolid (100%), vancomycin (100%), rifampicin (99.2%), cotrimoxazole (86%), tetracycline (84.4%) and gentamicin (83.6%). In this study, no isolate showed resistance to vancomycin and linezolid. The MRSA vancomycin and linezolid MICs ranged from 0.5 to 2 μg/ml and 1 to 2 μg/ml, respectively.

**Table 1:** Antibiotic susceptibility profiles of *S. aureus* isolates investigated in this study

Antibiotics	No. (%) MSSA (*n* = 68)			No. (%) MRSA (*n* = 54)			Overall resistance No. (%)	*p* value
	Susceptible	Intermediate	Resistant	Susceptible	Intermediate	Resistant		
Clindamycin	56 (82.4)	0 (0)	11 (16.2)^a^	27 (50)	0 (0)	4 (7.4)^b^	15(12.4%)	0.000^d^
Erythromycin	56 (82.4)	0 (0)	12 (17.6)	25 (46.3)	0 (0)	29 (53.7)	41(33.6)	0.000^d^
Gentamicin	57 (83.8)	1 (1.5)	10 (14.7)	45 (83.3)	3 (50)	6 (11.1)	16 (13.1)	0.401
Linezolid	68 (100)	0 (0)	0 (0)	54 (100)	0 (0)	0 (0)	0 (0)	–
Oxacillin	68 (100)	0 (0)	0 (0)	0 (0)	0 (0)	54 (100)	54 (44.3)	0.000^d^
Penicillin	22 (32.4)	0 (0)	46 (67.6)	0 (0)	0 (0)	54 (100)	100 (82.0)	0.000^d^
Rifampicin	68 (100)	0 (0)	0 (0)	53 (98.1)	0 (0)	1 (1.9)	1 (0.8)	0.260
Cotrimoxazole	56 (82.4)	0 (0)	12 (17.6)	49 (90.7)	0 (0)	5 (9.3)	17 (13.9)	0.184
Tetracycline	57 (83.8)	0 (0)	11 (16.2)	46 (85.2)	0 (0)	8 (14.8)	19 (15.6)	0.837
Vancomycin	68(100)	0 (0)	0 (0)	54 (100)	0 (0)	0 (0)	0 (0)	–
Ciprofloxacin	66 (97.1)	0 (0)	2 (2.9)	9 (16.7)	0 (0)	45 (83.3)	47 (38.5)	0.000^d^
Levofloxacin	67 (98.5)	0 (0)	1 (1.5)	9 (16.7)	0 (0)	45 (83.3)	46 (37.7)	0.000^d^
Moxifloxacin^c^	57 (98.3)	1 (1.7)	0 (0)	9 (16.7)	4 (7.4)	41(75.9)	41 (36.6)	0.000^d^
Cefoxitin	68 (100)	0 (0)	0 (0)	0 (0)	0 (0)	54 (100)	54 (44.3)	0.000^d^

^a^1 (1.5%) were inducible resistant; ^b^23 (42.6%) were inducible resistant; ^c^missing information for 10 MSSA isolates, ^d^ *p* < 0.05 indicates significant difference between MSSA and MRSA, –: not applicable

### Biofilm production of MRSA and MSSA isolates

Of the 122 *S. aureus* isolates tested, a majority (79.5%) were identified as strong biofilm producers. A total of 57 (83.8%) biofilm-producing isolates were MSSA and 41 (75.9%) isolates were MRSA (Table 2). In addition, 12.3% of *S. aureus* isolates were identified as moderate biofilm producers, 5.7% were identified as weak biofilm producers and 1.64% of strains did not produce biofilms.

**Table 2:** Distribution of biofilm and slime producers among MRSA and MSSA isolates

Biofilm/slime production	No. (%) isolates		*p* value*	
	MSSA (*n* = 68)	MRSA (*n* = 54)		
Biofilm				*p* = 0.241
Strong	57 (83.8)	41 (75.9)	*p* = 0.405	
Moderate	7 (10.3)	8 (14.8)		
Weak	4 (5.9)	3 (5.6)		
No biofilm	0 (0)	2 (3.7)		
Slime				
No slime	15 (22.1)	15 (27.8)	*p* = 0.19	
Intermediate	49 (72.1)	39 (72.2)		
Strong slime producer	4 (5.9)	0		

*p values were determined using *t* test, with *p* > 0.05 indicating no significant difference between groups

### Slime production of MRSA and MSSA isolates

Using Congo Red agar assay, most *S. aureus* isolates (72.1% MSSA and 72.2% MRSA isolates, respectively) were regarded as intermediate slime producers. There was no significant difference between MSSA and MRSA isolates in slime production (*p* = 0.19). Only 4 (6.0%) MSSA isolates demonstrated strong slime production after 24 h of incubation (Table 2).

### Distribution of biofilm-associated genes and agr/sar quorum sensing genes in MSSA and MRS isolates

In this study, the successful amplification of the 16S rRNA gene from all *S. aureus* isolates indicated the absence of PCR inhibitors in the bacterial DNA extracts. The amplification of biofilm-associated genes from MSSA and MRSA isolates using various singleplex and multiplex PCR assays is shown in Additional file 1: Fig. S1.

**Fig. 1:**
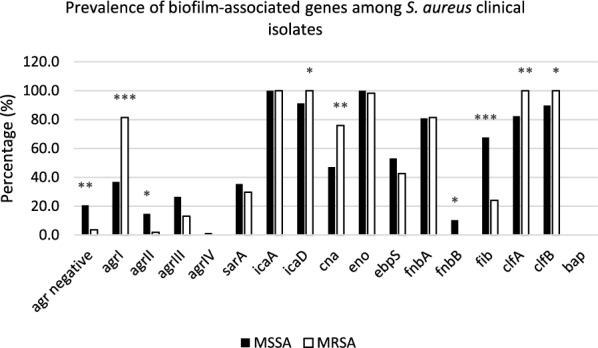
Prevalence of biofilm-associated genes among MSSA and MRSA clinical isolates. Paired *t* tests were performed to determine significant difference between MSSA and MRSA isolates. (*p < 0.05, **p < 0.01, ***p < 0.001)

The presence of the intracellular adhesion A (*icaA*) gene was observed in all *S. aureus* isolates (100%). There was variability in the distribution of other biofilm-associated genes in MSSA and MRSA (Fig. 1). Overall, the intracellular adhesion A and D (*icaA* and *icaD*), laminin-binding protein (*eno*), clumping factors A and B (*clfA* and *clfB*), and fibronectin-binding protein A (*fnbA*) were the most prevalent biofilm-associated genes in *S. aureus* isolates, regardless of MSSA or MRSA.

Compared to MSSA, the detection rates of *agrI, icaD, cna, clfA,* and *clfB* genes were significantly higher in MRSA, while the fibronectin-binding protein B (*fnbB*) gene was absent in all MRSA isolates. All MSSA isolates harbored the laminin-binding protein (*eno*) gene, while all MRSA isolates harbored intracellular adhesion D (*icaD)*, clumping factors A and B (*clfA* and *clfB*) genes. Intriguingly, the *bap* gene (encoding biofilm matrix protein) was not amplified from any of the isolates.

The number of biofilm-associated genes detected in *S. aureus* varied from three to eleven, with most isolates having 10 genes (including 24 MSSA and 10 MRSA isolates). However, the number of genes detected from an isolate was not significantly associated with biofilm production (*p* = 0.299, Pearson’s Chi-square, Table 3). Interestingly, the presence of *agrI* in MSSA (*p* = 0.018), and *ebpS* in MRSA isolates was significantly associated with biofilm production (*p* = 0.006) (Table 4).

**Table 3:** Cross-tabulation between the number of *S. aureus* biofilm-associated genes and biofilm production

Number of *S. aureus* biofilm-associated genes	Number of *S. aureus* isolates					
	Non-biofilm	Weak	Moderate	Strong	Total	*p* value
3	0	0	0	3	3	*p* = 0.299
4	0	0	0	3	3	
5	0	0	2	3	5	
6	0	0	2	1	3	
7	0	0	1	13	14	
8	0	2	3	22	27	
9	0	2	6	19	27	
10	3	2	1	28	34	
11	0	1	0	5	6	
Total	3	7	15	97	122	

*p value were determined using Pearson’s Chi-square

**Table 4:** Correlations between *agr, sarA*, and biofilm-associated genes, with biofilm production in 68 MSSA and 54 MRSA isolates investigated in this study

Genes	Biofilm production									
	No. MSSA isolates (*n* = 68)				*p* value	No. MRSA isolates (*n* = 54)				*p* value
	None	Weak	Moderate	Strong		None	Weak	Moderate	Strong	
*agrI*	0	4	1	20	0.018*	1	2	5	36	0.154
*agrII*	0	0	0	10	0.407	0	0	0	1	1.000
*agrIII*	1	0	3	14	0.175	1	1	2	3	0.090
*agrIV*	0	0	0	1	1.000	0	0	0	0	–
*sarA*	0	1	1	22	0.530	1	1	0	14	0.202
*icaA*	1	4	7	56	–	2	3	8	41	–
*icaD*	1	4	7	50	0.756	2	3	8	41	–
*cna*	1	1	3	27	0.740	2	3	6	30	0.635
*eno*	1	4	7	56	–	2	3	7	41	0.241
*ebpS*	1	3	4	28	0.782	2	3	5	13	0.006*
*fnbA*	1	4	4	46	0.304	2	2	5	35	0.307
*fnbB*	0	0	0	7	0.655	0	0	0	0	–
*fib*	1	3	5	37	1.000	1	2	2	8	0.267
*clfA*	1	4	5	46	0.633	2	3	8	41	–
*clfB*	1	4	6	50	1.000	2	3	8	41	–

*p < 0.05 indicates significant difference between groups; –: not applicable

In this study, the most prevalent *agr* type in *S. aureus* isolates was *agrI* (56.7%), followed by *agrIII* (20.5%) and *agrII* (9.0%). The *agrI* was detected with a significantly higher rate in MRSA (81.5%) as compared to MSSA (36.8%). In contrast, higher detection rates of *agrIII* and *agrII* were found in MSSA (26.5% and 14.7%, respectively) as compared to MRSA (13% and 1.9%, respectively). The *agrIV* was only detected in only one MSSA isolate (1.5%). Sequence analyses of representative *agr* alleles in this study demonstrated 100% similarity to *agrI* (352/352, 100%, GenBank accession no. AJ617710), *agrII* (472/472, 100%, GenBank accession no. AJ6177170), *agrIII* (333/333, 100%, GenBank accession no. AJ617723) and *agrIV* (577/577, 100%, GenBank accession no. AJ617712), as reported by Goerke et al. [33]. In this study, the presence of *agrI* was significantly correlated with ciprofloxacin (*p* = 0.000), levofloxacin (*p* = 0.003), moxifloxacin (*p* = 0.000), oxacillin (*p* = 0.000) and cefoxitin (*p* = 0.000) resistance (Table 5).

**Table 5:** Association between the presences of *agrI* with antibiotic susceptibility in *S. aureus* clinical isolates

Antibiotics	Susceptibility	No of isolates		*p* value
		*agrI*	Non *agrI*	
Clindamycin	S	41	42	0.002*
	R	7	8	
	IR	21	3	
Erythromycin	S	41	40	0.082*
	R	28	13	
Gentamicin	S	61	41	0.259
	R	7	9	
	IN	1	3	
Oxacillin	S	25	43	0.000*
	R	44	10	
Penicillin	S	10	12	0.342
	R	59	41	
Rifampicin	S	68	53	1.000
	R	1	0	
Cotrimoxazole	S	61	44	0.437
	R	8	9	
Tetracycline	S	64	39	0.005*
	R	5	14	
Ciprofloxacin	S	31	44	0.000*
	R	38	9	
Levofloxacin	S	31	45	0.000*
	R	38	8	
Moxifloxacin	S	27	39	0.000*
	R	35	6	
	IN	3	2	

Linezolid and vancomycin were excluded from the analysis as all *S. aureus* isolates were susceptible to these antibiotics. S: susceptible; R: resistant; IR: inducible resistant; IN: intermediate, *p < 0.05 indicates significant difference between groups

## Discussion

 The treatment and management of *S. aureus* infection pose significant challenges and a big threat in healthcare settings worldwide due to the emergence of antibiotic-resistant strains. In comparison with the Malaysia National Surveillance of Antimicrobial Resistance (NSAR) 2022 report [34], higher resistance rates to clindamycin (12.4% vs 5.9%), erythromycin (33.6% vs 9.9%) and gentamicin (13.1% vs 3.2%) were reported from a collection of clinical *S. aureus* isolates in this study. No linezolid-resistant strain was identified in this study, consistent with the very low percentage of linezolid resistance (0.4%) documented in the latest national report [34]. So far, the highest linezolid resistance rate was reported in a previous NSAR study (2010) whereby 7.7% in MRSA and 3.3% in MSSA were linezolid resistant [1], while there have been no studies documenting *S. aureus* resistance to vancomycin in Malaysia [35, 36].

In addition to antibiotic resistance, almost 80% of S. *aureus* isolates (MSSA and MRSA) in this study exhibited slime and biofilm production. However, no correlation was found between slime and biofilm production among staphylococcal isolates investigated in this study (Table 2). Similar observations have been reported for *S. aureus* human and animal isolates in earlier investigations [21, 37]. The lack of correlation between slime and biofilm production in *S. aureus* may be attributed to different measurement methods, i.e., Congo red agar method versus microtiter plate-based crystal violet assay, leading to disparities in the results. In addition, the complex nature of biofilm formation, possibly affected by bacterial genetic diversity, environmental factors, and regulatory mechanisms, may be attributed to the limited correlation between slime and biofilm production in *S. aureus.*

The most prevalent biofilm-associated genes detected in MRSA isolates in this study were intracellular adhesion A and D (*icaA* and *icaD*), laminin-binding protein (*eno*), clumping factors A and B (*clfA* and *clfB*), and fibronectin-binding protein A (*fnbA*), as shown in Fig. 1. The *agrI, icaD, cna, clfA,* and *clfB* genes were detected at significantly higher rates amongst MRSA isolates, while *fnbB* was detected at a significantly higher rate in MSSA isolates. The variability observed in the frequencies of biofilm-associated genes could be attributed to strain-to-strain difference [22, 38], source of isolation [39], and geographical settings [40]. Amongst the biofilm-associated genes, the elastin-binding protein (*ebpS)* gene has been significantly associated with biofilm production amongst MRSA isolates in this study (Table 3). Elastin-binding protein facilitates *S. aureus*-binding to elastin-rich tissues and promotes bacterial colonisation on mammalian tissues [41]. It has been significantly associated with strong biofilm production in *S. aureus* food isolates in two previous studies [38, 42].

The distribution of *agr* types is variable in *S. aureus* from different geographical regions [43]. In this study, the most prevalent *agr* type identified from *S. aureus* isolates was *agrI* (56.7%), followed by *agrIII* (20.5%) and *agrII* (9.0%), while *agrIV* (0.8%) has a low occurrence rate. Remarkably, a significantly higher percentage of MRSA isolates in this study was found to harbor *agrI*, compared to MSSA. The presence of *agrI* has been significantly associated with biofilm production amongst MSSA isolates in this study (Table 3), corresponding well with another study using nonclinical isolates [42]. Kawamura et al. [44] found that MRSA isolates haboring *agrII* have a significantly greater ability to produce biofilm, however; Usun Jones et al. [21] and Cha et al. [45] found no variation in MRSA biofilm production among different *agr* groups. The difference might be attributed to variations between strains, potentially resulting from microbial adaptation and geographical influences.

As the transcription of the *agr* locus (I–IV) is auto-inducing peptide (AIP)-dependent, the differentiation of staphylococcal strains based on *agr* typing may provide further insights into the epidemiology and antibiotic resistance. Studies have shown that the *mecA* gene of MRSA indirectly activates AIPs which significantly affect biofilm production, quorum-sensing and virulence, and antibiotic resistance [17, 18]. As quorum sensing is higly influenced by cell density, high-density colonies can produce numerous small molecule signals, triggering downstream processes, such as virulence and antibiotic resistance mechanisms, which poses a threat to the host and antibiotic efficacy [46]. Biofilm production has been reported to provide a niche for generation of antibiotic resistant subpopulations or persister cells through the exchange of genetic materials [47]. Recent data demonstrated a significant correlation between *agr*I with cefoxitin and erythromycin resistance [48], as well as tetracycline, erythromycin, clindamycin, and ciprofloxacin resistance in *S. aureus* [43]. Interestingly, a significant association was found between *agrI* with fluoroquinolones (ciprofloxacin, levofloxacin, and moxifloxacin) resistance (*p* < 0.05) for the first time in this study. In addition, the high resistance (75.9%) of MRSA against fluoroquinolones especially moxifloxacin, a fourth-generation fluoroquinolone, is alarming (Table 1).

Fluoroquinolone exposure has been identified as an increased risk factor for MRSA isolation and infection [49–51]. The key mechanims to *S. aureus* fluoroquinolone resistance are through chromosomal point mutations in *gyrA/B* (DNA gyrase subunits), *grlA/B* (DNA topoisomerase IV subunits), and the promoter region of *norA* efflux pump [52]. The accumulation of such mutations may be enhanced in biofilm producing *agr1*-habouring strains, contributing to a high level of resistance to fluoroquinolones, as observed in the MRSA isolates in this study. However, more extensive studies are required to explore the linkage between *agr1*, biofilm production and fluoroquinolone resistance.

One of the limitation of this study is its confinement to a single-center setting and convenient sampling of *S. aureus* isolates, thus the ratio of MSSA to MRSA might not reflect the actual prevalence of multidrug resistant *S. aureus* in the local setting. For more comprehensive insights, future studies are recommended to include diverse sampling methods and multiple centers, to ensure a more representative analysis of the genetic diversity and prevalence of biofilm-associated genes in the Malaysian isolates. As the antibiotic susceptibility profiling of *S. aureus* isolates was limited to planktonic cells, future reserach should also include comprehensive assessment of antibiotic susceptibility within biofilm structures to enhance understanding of their impact on biofilm-associated *S. aureus* infections. In addition, the utilization of *mec* (SCCmec) typing would be beneficial for identifying distinct MRSA types and establishing correlations with other study variables. As conventional antibiotics do not work effectively against *S. aureus* biofilm infection, new therapeutic strategies and infection control practices are urgently needed. The genetic profiling of biofilm-associated genes and quorum sensing systems of *S. aureus* isolates has provided scientific foundation for developing a more targeted approach for surveillance, and treatment against biofilm infection in our clinical setting.

## Conclusion

The emergence of multidrug-resistant *S. aureus* strains has been driven by the use of multiple antibiotic classes over the years. The high rates of resistance against clindamycin, erythromycin, and fluoroquinolones as reported in this study have called for more judicious use of antibiotics for treatment of MRSA infection in this region. More importantly, the identification of prevalent biofilm-associated genes and *agr* types associated with antibiotic resistance in this study has shed valuable genetic insights into *S. aureus* biofilm formation, which are important to tailor more focused surveillance and treatment strategies against *S. aureus* biofilm infection in our setting.

### Supplementary Information

**Additional file 1: Table S1.** Source of *S. aureus* clinical isolates. **Table S2.** Nucleotide sequences of primers and thermal cycling conditions used in this study. **Table S3.** MIC range, MIC_50_ and MIC_90_ values of 112 *S. aureus* isolates against various classes of antibiotics. **Figure S1.** Agarose gel electrophoresis results for amplified biofilm associated gene fragments of *S. aureus*.

## Data Availability

The data sets used and/or analysed during the current study are available from the corresponding author on reasonable request.

## Abbreviations

AIP: Auto-inducing peptide
CFU: Colony forming unit
CLSI: Clinical Laboratory Standard Institute
MIC: Minimum inhibitory concentration
MIC_50_: Lowest concentration of the antibiotic at which 50% of the isolates were inhibited
MIC_90_: Lowest concentration of the antibiotic at which 90% of the isolates were inhibited
MRSA: Methicillin-resistant *Staphylococcus aureus*
MSSA: Methicillin-susceptible *Staphylococcus aureus*
OD: Optical density
*S. aureus*: *Staphylococcus aureus*
UMMC: Universiti Malaya Medical Centre
