# Supplementary material for: Unveilling genetic profiles and correlations of biofilm-associated genes, quorum sensing, and antibiotic resistance in Staphylococcus aureus isolated from a Malaysian Teaching Hospital
Source: Eur J Med Res. 2024 Apr 22;29:246. doi: 10.1186/s40001-024-01831-6 (PMC11036768; doi:10.1186/s40001-024-01831-6)
Supplement: Supplementary file 1 — Additional file 1: Table S1. Source of S. aureus clinical isolates. Table S2. Nucleotide sequences of primers and thermal cycling conditions used in this study. Table S3. MIC range, MIC50 and MIC90 values of 112 S. aureus isolates against various classes of antibiotics. Figure S1. Agarose gel electrophoresis results for amplified biofilm associated gene fragments of S. aureus. [file 40001_2024_1831_MOESM1_ESM.docx]

**Table S1.** **Source of** *S. aureus* **clinical isolates**

| **Types of specimens** | **No.(%) isolates** | | |
| --- | --- | --- | --- |
|  | **MSSA (n=68)** | **MRSA**  **(n=54)** | **Overall (n=122)** |
| Blood | 18 (26.5) | 20 (37) | 38 (31.1) |
| Sterile tissues | 22 (32.4) | 14 (25.9) | 36 (29.5) |
| Pus | 11 (16.2) | 4 (7.4) | 15 (12.3) |
| Wound swab | 6 (8.8) | 5 (9.3) | 11 (9) |
| Lower respiratory tract specimen | 9 (13.2) | 9 (16.7) | 18 (14.8) |
| Others | 2 (2.9) | 2 (3.7) | 4 (3.3) |

**Table S2. Nucleotide sequences of primers and thermal cycling conditions used in this study**

| **Genes** | **Primers** | | **Nucleotide sequence (5’ – 3’)** | | **Fragment size (bp)** | | **Annealing temperature (ºC)** | |
| --- | --- | --- | --- | --- | --- | --- | --- | --- |
| 16SrRNA | 27F  1492R | | AGAGTTTGATCCTGGCTCAG TACGGTTACCTTGTTACGACTT | | 1500 | | 54 | |
| *sarA* | *sarAF*  *sarAR* | | CCCAGAAATACA ATCACTGTG  AGTGCCATTAGTGCAAAACC | | 720 | | 53 | |
| *agr I* | *pan-agr*  *agr I* | | ATGCACATGGTGCACATGC  GTCACAAGTACTATAAGCTGCGAT | | 440 | | 55 | |
| *agr II* | *pan-agr*  *agr II* | | ATGCACATGGTGCACATGC  GTATTACTAATTGAAAAGTGCCATAGC | | 572 | | 55 | |
| *agr III* | *pan-agr*  *agr III* | | ATGCACATGGTGCACATGC  CTGTTGAAAAAGTCAACTAAAAGCTC | | 406 | | 55 | |
| *agr IV* | *pan-agr*  *agr IV* | | ATGCACATGGTGCACATGC  CGATAATGCCGTAATAC CCG | | 588 | | 55 | |
| *bap* | Forward  Reverse | | CCCTATATCGAAGGTGTAGAATTG  GCTGTTGAAGTTAATACTGTACCTGC | | 971 | | 62 | |
| *ebpS* | *EBP-1*  *EBP-2* | | CATCCAGAACCAATCGAAGAC  CTTAACAGTTACATCATCATGTTTATCTTTG | | 652 | | 55 | |
| *eno* | *ENO-1*  *ENO-2* | | ACGTGCAGCAGCTGACT  CAACAGCATYCTTCAGTACCTTC | | 302 | | 55 | |
| *fib* | *FIB-1*  *FIB-2* | | CTACAACTACAATTGCCGTCAACAG  GCTCTTGTAAGACCATTTTCTTCAC | | 404 | | 55 | |
| *fnbA* | *FNBA-1*  *FNBA-1* | | GTGAAGTTTTAGAAGGTGGAAAGATTAG  GCTCTTGTAAGACCATTTTTCTTCAC | | 127 | | 55 | |
| *fnbB* | *FNBB-1*  *FNBB-2* | | GTAACAGCTAATGGTCGAATTGATACT  CAAGTTCGATAGGAGTACTATGTTC | | 524 | | 55 | |
| *clfA* | *CLFA-1*  *CLFA-2* | | ATTGGCGTGGCTTCAGTGCT  CGTTTCTTCCGTAGTTGCATTTG | | 292 | | 55 | |
| *clfB* | *CLFB-1*  *CLFB-2* | | ACATCAGTAATAGTAGGGGGCAAC  TTCGCACTGTTTGTGTTTGCAC | | 205 | | 55 | |
| *cna* | | *CNA-2*  *CNA-1* | | GTCAAGCAGTTATTAACACCAGAC  AATCAGTAATTGCACTTTGTCCACTG | | 423 | | 55 |
| *icaD* | *icaDF*  *icaDR* | | ATG GTC AAG CCC AGA CAG AG  CGT GTT TTC AAC ATT TAA TGC AA | | 188 | | 55 | |
| *icaA* | *icaAF*  *icaAR* | | ACA CTT GCT GGC GCA GTC AA  TCTGGAACCAACATCCAACA | | 198 | | 55 | |

**Table S3. MIC range, MIC_50_ and MIC_90_ values of 112 *S. aureus* isolates against various classes of antibiotics**

| **Antibiotic** | **Antibiotic classes** | **MIC range (µg/mL)** | | **MIC_50_ (µg/mL)** | | **MIC_90_ (µg/mL)** | |
| --- | --- | --- | --- | --- | --- | --- | --- |
|  |  | **MSSA** | **MRSA** | **MSSA** | **MRSA** | **MSSA** | **MRSA** |
| Clindamycin | Lincosamides | 0.25 - 8 | 0.25 – 8 | 0.25 | 0.25 | 0.25 | 8 |
| Erythromycin | Macrolides | 0.25 - 8 | 0.25 – 8 | 0.25 | 0.5 | 0.25 | 8 |
| Gentamicin | Aminoglycosides | 0.5 - 8 | 0.5 – 16 | 0.5 | 0.5 | 0.5 | 8 |
| Linezolid | Oxazolidinones | 1 - 2 | 1 - 4 | 2 | 2 | 2 | 2 |
| Oxacillin | Penicillinase-stabile penicillins | 0.25 - 1 | 4 - 4 | 0.5 | 4 | 0.5 | 4 |
| Penicillin | Penicillinase-labile penicillins | 0.03 - 0.5 | 0.5 - 0.5 | 0.5 | 0.5 | 0.5 | 0.5 |
| Rifampicin | Ansamycins | 0.25 – 0.5 | 0.5 – 32 | 0.5 | 0.5 | 0.5 | 0.5 |
| Cotrimoxazole | Sulfonamides | 10 - 320 | 10 - 320 | 10 | 10 | 10 | 10 |
| Tetracycline | Tetracyclines | 1-16 | 1-16 | 1 | 1 | 16 | 16 |
| Vancomycin | Glycopeptides | 0.5 - 2 | 0.5 - 1 | 1 | 0.5 | 1 | 1 |
| Ciprofloxacin | Fluoroquinolones | 0.5 - 8 | 0.5 – 8 | 0.5 | 8 | 0.5 | 8 |
| Levofloxacin | Fluoroquinolones | 0.12 - 4 | 0.12 – 8 | 0.25 | 8 | 0.25 | 8 |
| Moxifloxacin* | Fluoroquinolones | 0.25 - 1 | 0.25 – 4 | 0.25 | 2 | 0.25 | 2 |

*Data for 10 isolates was not available


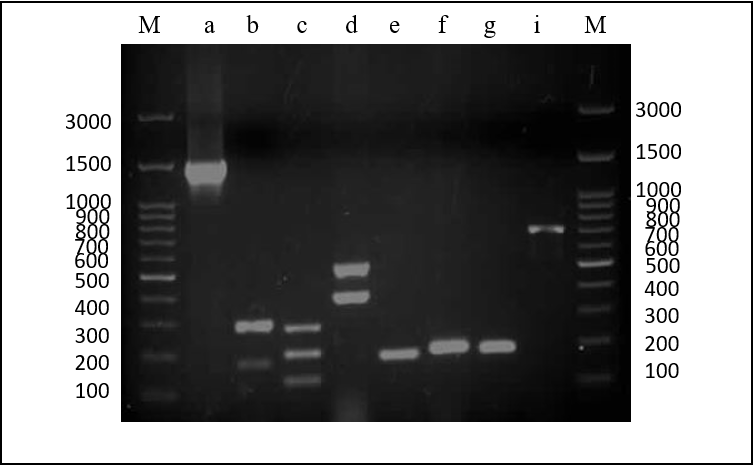


**Figure S1. Agarose gel electrophoresis results for amplified biofilm associated gene fragments of *S. aureus*.**

(M) 100 bp DNA molecular marker (Simply, Taiwan); (a) 16S rRNA (1500 bp); (b) Multiplex 1 – *eno* (302bp), *ebpS* (186 bp); (c) Multiplex 2 – *clfA* (292bp), *clfB* (205 bp), *fnbA* (128 bp); (d) Multiplex 3 – *fnbB* (524bp), *fib* (404 bp); (e) *icaA* (188 bp); (f) *icaD* (198 bp); (g) *cna* (192 bp); (i) *sarA* (720bp).
